# Idiopathic multicentric castleman’s disease mimicking immunoglobulin G4-related disease responding well to Bortezomib: a case report

**DOI:** 10.1186/s12882-023-03335-7

**Published:** 2023-10-02

**Authors:** Qian Peng, Fan Wu, Yuting Shi, Juan Wang, Zhimin Zhai, Zhitao Wang

**Affiliations:** 1 Department of Hematology/Hematological Lab, Second Hospital of Anhui Medical University, Hefei, 230601 China; 2 Department of Radiology, Second Hospital of Anhui Medical University, Hefei, 230601 China

**Keywords:** Castleman disease, IgG4-related disease, PET/CT, Histopathological features, Case report

## Abstract

**Background:**

Castleman’s disease (CD) is a rare disease that has clinical and pathological similarities to lymphoma and is characterized by a high frequency of associated immunological dysfunction. ImmunoglobulinG4-related disease (IgG4-RD) is a collection of systemic disorders that affect numerous organs and are also referred to as IgG4-associated sclerosing diseases. CD and IgG4-RD are difficult to separate because they may manifest similar commin clinical features.

**Case presentation:**

This case describes a 53-year-old female who, during routine medical check-up, exhibited a progressive increase in serum globulin levels and a simultaneous worsening of anemia symptoms, raising concern for a clonal plasma cell disease such as myeloma. However, bone marrow punctures did not reveal any abnormal plasma cells. Also, serum and urine immunofixation electrophoresis demonstrated no abnormal monoclonal protein bands. In addition, several laboratory findings excluded chronic liver disease, chronic infections caused by bacteria or viruses. Later, we found elevated serum IgG4 levels (10,700 mg/L), and identified multiple enlarged lymph nodes throughout the patient’s body. Axillary lymph node aspiration revealed no abnormal lymphocytes, ruling out the possibility of lymphoma. Pathological morphology of the axillary lymph revealed a large number of plasma cells in the lymphatic follicles. In addition, there was a reduction in lymphatic follicle size and apoptosis of the germinal centres. Immunohistochemistry revealed IgG4+/IgG + in > 40% of cells, and more than 100 IgG4 + cells per high powered field (HPF) of specimen. As of now, finding strongly suggested IgG4-RD. This patient was treated with glucocorticoids and various immunosuppressive drugs, such as prednisone, cyclosporine, methotrexate, cyclophosphamide, mycophenolate mofetil, azathioprine and hydroxychloroquine. Unfortunately, the patient did not recover. Ultimately, idiopathic multicentric Castleman disease (iMCD) was diagnosed in relation to the patient’s clinical presentation and laboratory tests, and after combination chemotherapy (VCD: Bortezomib, Cyclophosphamide and Dexamethasone), durable remission was achieved without serious adverse effects. During the follow-up period of one year and ten months, the patient remained stable.

**Conclusion:**

The diagnosis of Castleman must be distinguished from other disorders such as IgG4-RD, malignant lymphoma, reactive hyperplasia of various lymph nodes (mostly caused by viral infections), plasmacytoma, advanced HIV and rheumatic diseases. Besides observing systemic symptoms, laboratory tests such as immunoglobulin levels, complement levels, interleukin levels, and C-reactive protein levels should also be performed in order to determine a diagnosis.

## Background

Benjamin Castleman described a clinically rare benign lymphoproliferative condition in 1954, called Castleman disease (CD), characterized as either unicentric Castleman disease (UCD) or multicentric Castleman disease (MCD), depending on the extent of lymph node involvement [1, 2]. There are two histological types of Castleman disease, the hyaline vascular and plasma cell variants, or, less commonly, a mixed type of Castleman disease.

The plasma cell type is characteristic of most cases of MCD, which are frequently accompanied by systemic inflammation, fever, anemia, diffuse lymphadenopathy, cytopenia, and even life-threatening multi-organ dysfunction [3]. The majority of MCD cases may be associated with dual infection involving human herpesvirus-8 (HHV8) and HIV [4, 5]. In such cases, almost all patients present with splenomegaly, oedema, effusion, respiratory symptoms or haemophagocytic syndromes. Additionally, more than half of these patients develop Kaposi sarcoma [6]. Laboratory abnormalities are also common, including anemia, thrombocytopenia, high serum CRP, low serum albumin and hyperglobulinemia [4, 7, 8]. On the other hand, some MCD cases are HHV-8 negative and unrelated to HIV infection, primarily classified as idiopathic MCD (iMCD).

In 2012, an international multidisciplinary study group approved the term ‘IgG4-RD’ that describes a fibro-inflammatory disorder with elevated serum IgG4 levels and plasma cells with IgG4 + staining in inflammatory lesions [9, 10]. Differentiating IgG4-RD from MCD is challenging because both diseases present high serum IgG4. In this article, we present the case of a patient who was difficult to diagnose and who had no recurrence for two years after being treated.

### Case presentation

**Fig. 1:**
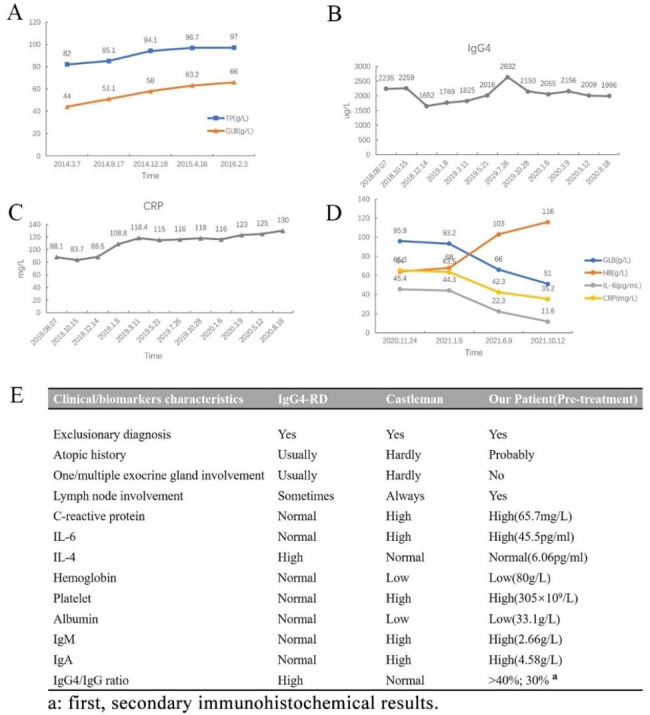
(**A**) During the asymptomatic period, the patient has a progressive increase in serum TP and GLB; (**B**) Persistently high serum CRP since onset; (**C**) Serum IgG4 levels still have not reduced after two years of hormone and immunosuppressive therapy; (**D**) After chemotherapy, the patient’s serum HB, GLB, IL-6 and CRP all returned to a normal level; (**E**) Description of the patient’s clinical features and biomarkers

In October 2013, an asymptomatic 46-year-old female was found to have elevated total protein (TP:99.3 g/L) and globulin (GLB:63.6 g/L) during laboratorial investigation. At the same hospital outpatient clinic, immunoglobulin levels (IgG 31.03 g/L, IgA 4.58 g/L, IgM 2.66 g/L) were also elevated. However, peripheral blood immunophenotyping by flowcytometry (CD38 + CD56-CD45dim:0%), the levels of anti-nuclear antibody and immunofixation electrophoresis were all within normal limits. The patient refused admission to hospital, and over the next two years, regular follow-up examinations revealed a gradual increase in TP and GLB levels, but still no symptoms (Fig. 1A).

**Fig. 2:**
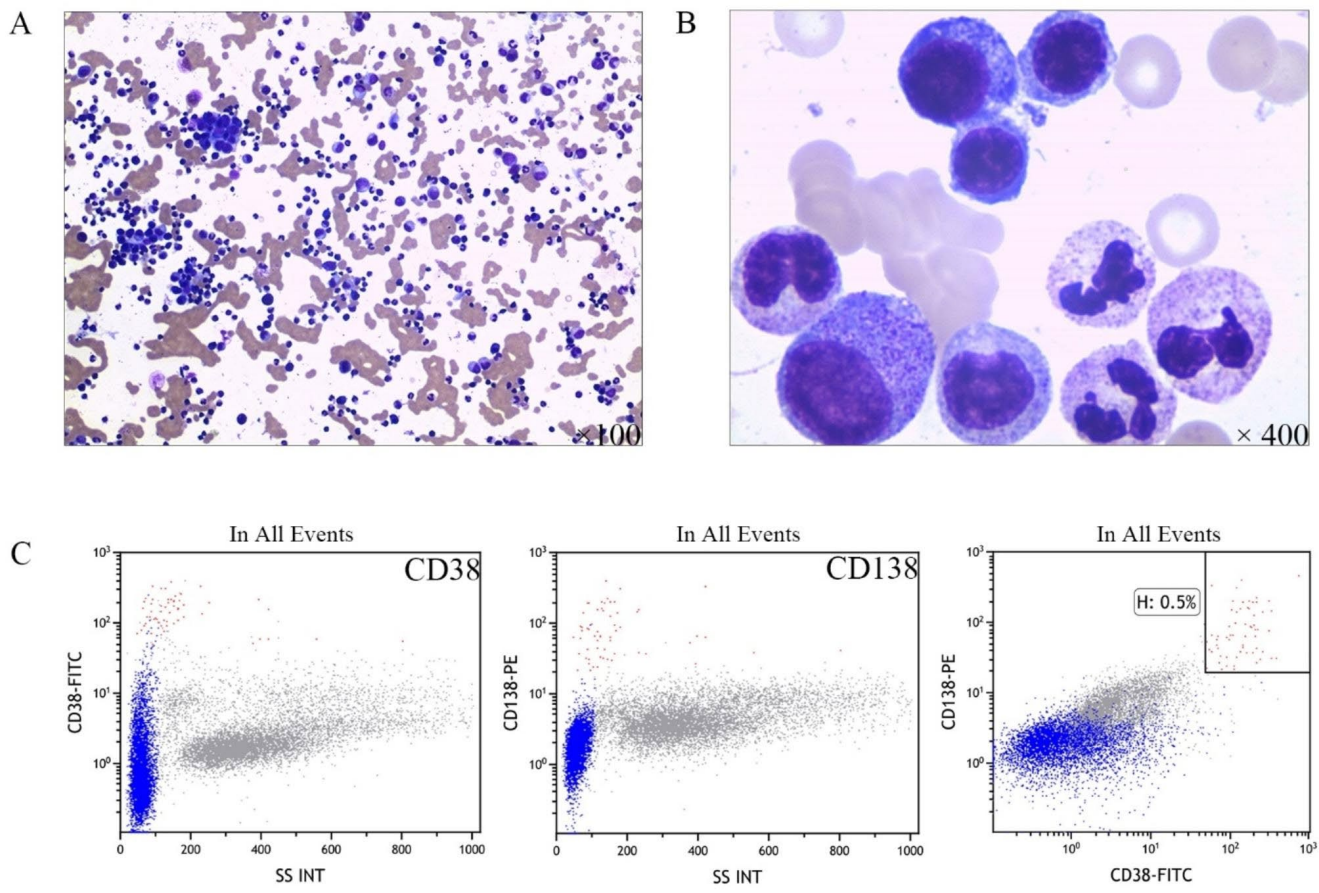
(**A**, **B**) Cytology of bone marrow aspiration shows no abnormal plasma cells (low magnification: ×100; high magnification: ×400); (**C**) Flow immunophenotyping shows CD38 + CD138 + plasma cells

In the third year, the patient developed weakness, anemia and enlarged lymph nodes. On lymph node puncture, no abnormal lymphocytes were observed. On bone marrow cell morphology (Fig. 2A and B) and immunophenotyping (CD38 + CD138+:0.5%) (Fig. 2C), no clonal plasma cells were observed. Laboratory tests revealed elevated cytokines such as IL-6 (45.4pg/mL) and TNF-α (14.6pg/mL), as well as elevated various IgG subtypes (IgG1:41600 mg/L, IgG2:17000 mg/L, IgG4: 10,700 mg/L). During the four years from 2013 to 2017, multiple results suggested polyclonal hyperglobulinemia, a progressive decrease in hemoglobin, a progressive increase in TP and GLB, consistently high levels of CRP and ESR, and a lack of other unusual results (both HHV8 and HIV were negative).

In August 2018, the patient presented with recurrent fever and a rash with pruritus. IgG4 remained high and the final diagnosis of IgG4-RD was made in conjunction with medical history. Over the next two years, the patient was treated with prednisone and various immunosuppressive drugs, but while the white blood cell count and platelet count remained stable, the anemia worsened progressively, CRP increased progressively, and IgG4 did not decrease significantly (Fig. 1B and C). The patient responded poorly to treatment, and developed fevers and rashes more frequently.

**Fig. 3:**
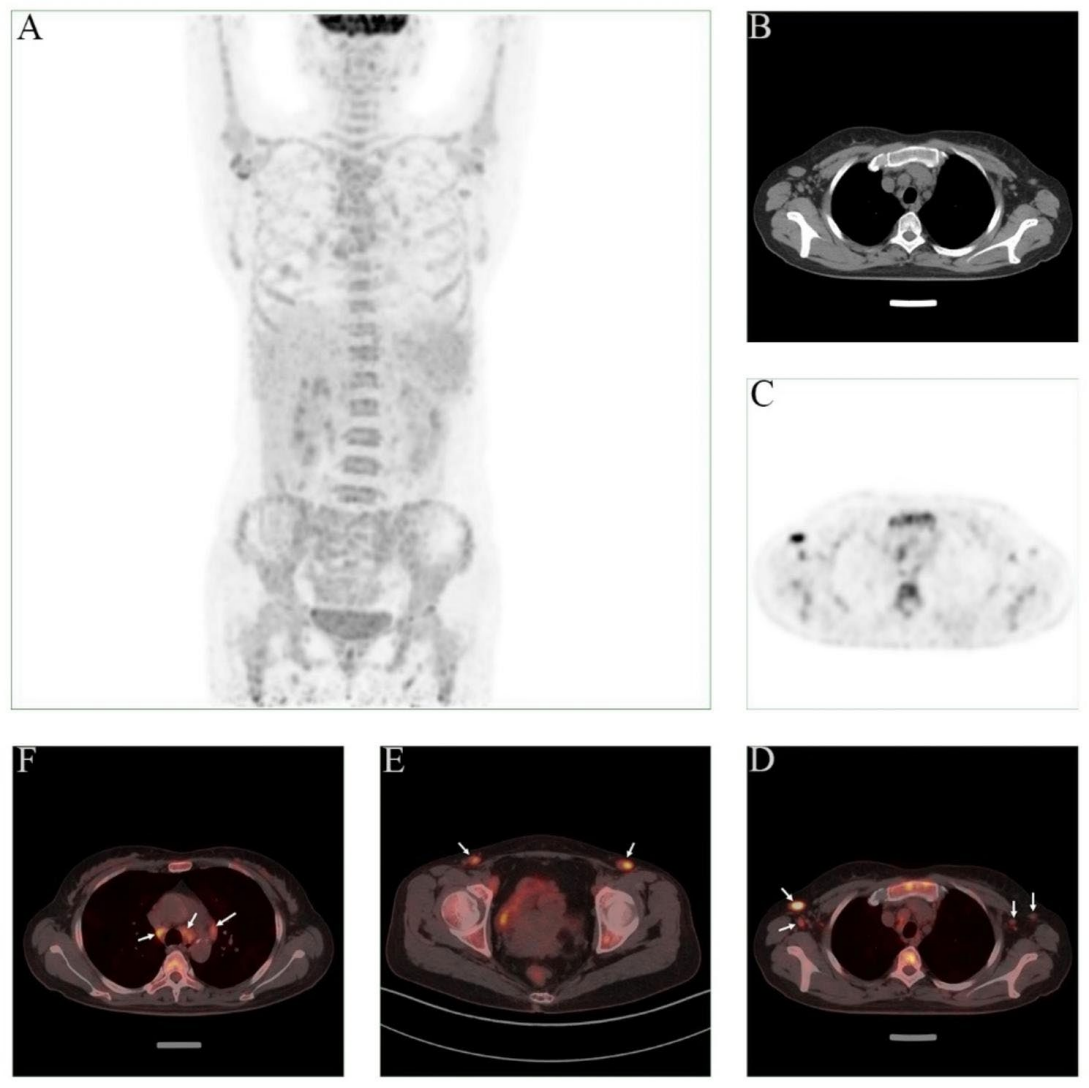
(**A**) ^18^ F-FDG PET/CT images showing diffuse elevated ^18^ F-FDG metabolism in bone and multiple lymph nodes throughout the body; (**B**-**D**) PET/CT images of axillary lymph nodes showed high uptake of 18 F-FDG, suggesting increased metabolism; (**E**, **F**) Bilateral mediastinal pleura, and inguinal lymph nodes all showed increased metabolism

**Fig. 4:**
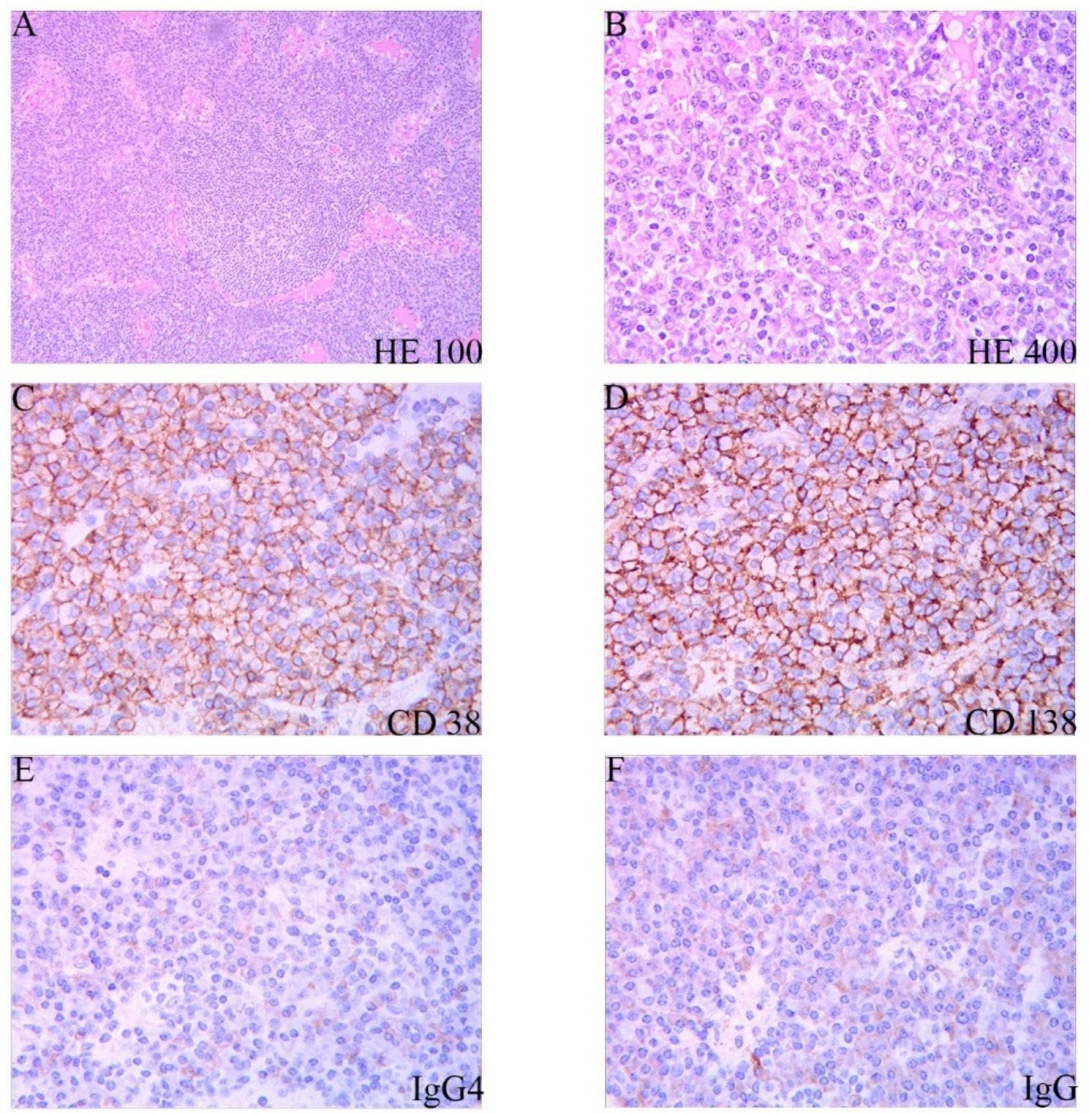
(**A**) At low magnification, the lymphatic sinuses are open, the lymphatic follicles are visible, the germinal centres are narrowed, and numerous mature plasma cell infiltrates are seen between the follicles in a lamellar pattern (×100); (**B**) The infiltrating plasma cells are cytoplasm-rich, dichromatophilic, with large round, deviated nuclei and wheel-like chromatin under high magnification (×400); (**C**, **D**) CD38 and CD138 immunohistochemical staining showed a large number of positive plasma cells between the follicles; (**E**) Immunoglobulin (IgG) positive plasma cells could be seen by immunohistochemical staining; (**F**) IgG4-positive plasma cells could be seen by immunohistochemical staining; The IgG4/IgG ratio was greater than 40%

In November 2020, this patient was referred to our department, and lab tests were performed as before (Hb:64 g/L, TP:113.4 g/L, GLB:95.9 g/L, CRP:75.3 mg/L, IgG: 47.36 g/L, IgA:5.92 g/L, IgM:2.78 g/L). The patient currently suffers from severe hyperglobulinemia persisted with high levels of serum IgG (IgG1:35.1 g/L, IgG2:15.1 g/L, IgG3:30.4 g/L, IgG4:12.7 g/L). Fluorodeoxyglucose positron emission tomography (FDG-PET) indicated diffuse increased 18 F-FDG metabolism in bone throughout the body, splenomegaly with increased 18 F-FDG metabolism, multiple lymph nodes of variable size in the bilateral neck, submandibular, mediastinal, axillary, bilateral hilar, peritoneal, retroperitoneal, bilateral parietal iliac vessels and inguinal areas with increased 18 F-FDG metabolism (Fig. 3A-F). A biopsy of the right axillary lymph node revealed a largely preserved lymph node structure with reduced lymphoid follicles and germinal center atrophy. In addition, a large number of plasma cells were found to be infiltrating the area. Immunohistochemistry showed scattered B cells (CD20+) and T cells (CD3, CD4+) with increased plasma cells (CD38+, CD138+, CD56 negative, Pax5 negative, Ki67 approximately 15%, MUM1+, EMA+, Cyclin D1 negative, Kappa+, Lambda+, IgG+), IgG4 positive cells > 100/HPF, and IgG4/IgG > 40%, in situ hybridization: EBER (-) (Fig. 4A-F). On this basis, a diagnosis of IgG4-RD was still favoured. However, based on the patient’s pre-treatment clinical features and laboratory findings (Fig. 1E), as well as seven-year treatment history (ineffectiveness following IgG4-RD treatment protocols) and continuously changing clinical presentation, a diagnosis of multicentric Castleman disease appears to be more fitting for the patient. In addition, an external biopsy of the right axillary lymph node was sent to another hospital. Immunohistochemistry showed CD38 and CD138 (plasma cells) (multiple positive), MUM1 (+), Kappa (multiple positive), Lambda (multiple positive), CMV (+), Ki67 (< 5%+), CD20 (+), PAX (+), CD3 and CD4 were scattered (T cells) (+), IgG (+), IgG4 (30%+), in situ hybridization: EBER (-). It was observed that the immunohistochemistry results were similar to those of our first examination at our hospital, except that the IgG4/IgG ratio was 40% lower. The patient was again diagnosed with multicentric Castleman disease (plasma cell type, iMCD).

**Fig. 5:**
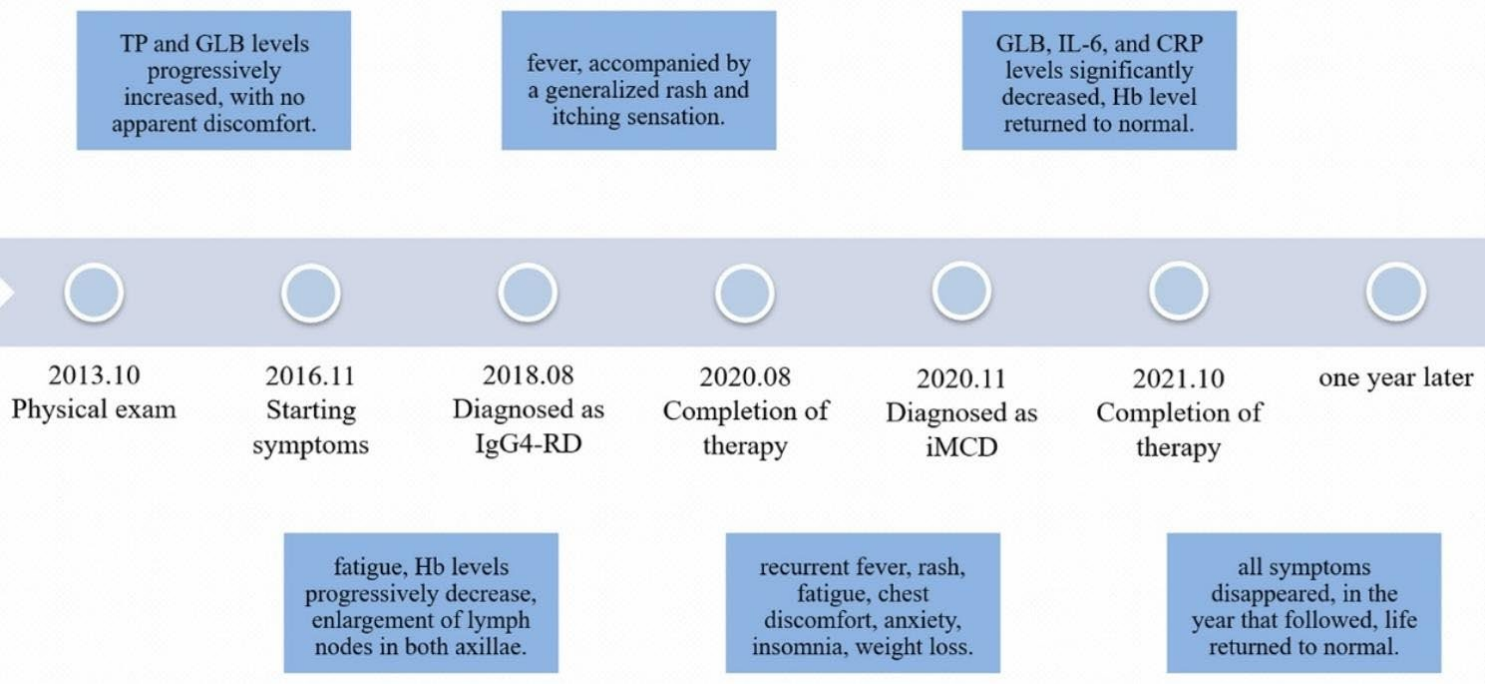
The disease history of the patient

The diagnosis of iMCD was confirmed upon the presentation of these histopathological findings and clinical features. Due to the inadequate response to previous glucocorticoid treatment, we chose to use a bortezomib-based chemotherapy regimen. The patient received seven VCD cycles (VCD: bortezomib, cyclophosphamide, dexamethasone) between November 2020 and October 2021. Following the completion of the full treatment regimen in October 2021, the patient’s serum levels of GLB, IL-6, and CRP decreased significantly and Hb returned to normal (Fig. 1D). Subsequent follow-up one month after the last treatment revealed that the patient’s symptom, including fatigue, fever, and rash had completely resolved, and the enlarged lymph nodes had disappeared. Over the course of the following year, the patient did not require hospitalization or treatment for any symptoms and was followed up via telephone. During this period, there was substantial improvement in her mood. A brief introduction of disease history was presented in Fig. 5.

## Discussion and conclusion

Known as a rare polyclonal lymphoproliferative disorder, iMCD is characterized by lymphadenopathy and inflammatory symptoms. Symptoms of systemic disease and cytopenia are the most common presentation features. It is possible to have enlarged lymph nodes in iMCD at any lymph node station. Other tissues may also be involved, accompanied by elevated levels of serum IgG4 and IgG4 + plasma cell infiltration [11].

Furthermore, over half of iMCD cases are associated with renal involvement [12, 13], such as thrombotic microangiopathy (TMA)-like renal lesions, amyloidosis, membranoproliferative glomerulonephritis, mesangial proliferative glomerulonephritis, crescentic glomerulonephritis. In fact, they represent a clinical subtype of iMCD known as iMCD-TAFRO, characterized by thrombocytopenia, ascites, reticulin fibrosis, renal dysfunction, organomegaly. In our case, the absence of renal dysfunction, thrombocytopenia, and organomegaly with hypergammaglobulinemia classifies it as iMCD-not otherwise specified (iMCD-NOS). The former is more challenging to differentiate from systemic lupus erythematosus (SLE) and myelofibrosis, while the latter is more difficult to distinguish from autoimmune lymphoproliferative syndrome and IgG4-RD [14].

A newly recognized fibroinflammatory disease of the 21st century, IgG4-RD is characterized by elevated serum IgG4 levels, plasma cell infiltration, fibrosis in the affected tissues, hyperplasia and enlargement of the involved organs, as well as symptoms like obstruction or compression. Clinical manifestations can cause one or more organs to be affected, resulting in glucocorticoid therapy having a favorable effect, but recurrence is a potential problem. IgG4-RD is diagnosed by (i) clinical signs of diffuse or characteristic enlargement, tumors, nodules, or hypertrophy of an organ or multiple organs, (ii) serum IgG4 + > 135 mg/dl, or (iii) high levels of IgG4 + plasma cells (> 40% of total IgG + plasma cells, > 10 IgG4 + plasma cells per high-power field). The presence of two or more of the above factors may lead to the diagnosis of IgG4-RD [10, 15].

Both share the common feature of dense lymphoplasmacytic infiltrates and a high proportion of IgG4-bearing plasma cells with a high serum IgG4 level. IgG4-RD shares similarities with Castleman disease, however, there are also differences: (i) Systemic symptoms: IgG4-RD commonly accumulates in multiple glandular organs such as the parotid, lacrimal and salivary glands and the pancreas, and it can occur alone or in association with IgG4-related disease in other organs [16–18]. iMCD patients have a number of systemic symptoms including anemia, weakness, several enlarged lymph nodes and weight loss. (ii) Hypergammaglobulinemia: In addition to elevated IgG, patients with iMCD also have elevated IgM and IgA. When compared to iMCD, IgG4-RD tend to have higher levels of IgG4 and a higher IgG4/IgG ratio. Conversely, IgA, IgM, IgG1 and IgG1/IgG ratios are observed to be lower in IgG4-RD patients. (iii) Laboratory tests: In patients with iMCD, usually, globulins are progressively elevated and HB progressively decreases with persistent elevations in inflammatory indicators such as CRP and IL-6. (iv) Pathologic features: IgG4-RD is characterized by polyclonal lymphoplasmacytic infiltrates with high IgG4 + plasma cell counts (> 40% of total IgG + plasma cells) and fibrosis, but this ratio is often lower than 40% in iMCD patients. (v) Treatment results: IgG4-RD demonstrates an initial favorable response to corticosteroid therapy. Nearly half of iMCD patients exhibit some transient improvement after receiving high-dose corticosteroid treatment, although it may not completely suppress symptoms or signs, and relapses can occur.

This patient has recurrent fever and rash with generalized symptoms such as malaise, chest tightness, anxiety, insomnia and lethargy, no other glandular involvement, no sensitivity to glucocorticoids or immunosuppressive drugs, and markedly elevated serum IL-6, CRP, IgA, IgM, IgG (IgG1, IgG2, IgG3, IgG4). Despite the fact that the pathology supported the diagnosis of IgG4-RD, the IgG4+/IgG + plasma cell ratio had just exceeded the 40% threshold. Interestingly, the ratio of IgG4+/IgG + plasma cell decreased dramatically when tested again at another hospital, and as a result, the 40% cut-off point cannot be used with certainty as a gold standard for differentiating iMCD from IgG4-RD, but rather only as a proposal [19]. IgG4-RD is a diagnosis of exclusion, because it is difficult to distinguish between iMCD and IgG4-RD on the basis of pathomorphology or even immunohistochemistry alone, and it is necessary to combine the patient’s clinical presentation, laboratory tests, atopic history, levels of IgA and CRP to make a definitive diagnosis and provide a basis for clinical treatment and prognostic assessment.

When selecting treatment for iMCD patients, the severity of the disease must be taken into consideration. However, regardless of disease severity, first-line treatment should primarily focus on anti-IL-6 directed therapy [14, 20]. For patients with a clear diagnosis and milder disease, treatment is with siltuximab/tocilizumab +/- steroids. For more severe disease siltuximab/tocilizumab and higher dose steroids. Patients are classified as severe when they present with systemic oedema, ascites, ECOG PS ≥ 2, eGFR < 30 min/mL, Hemoglobin ≤ 8.0 g/dL, and current manifestations of pulmonary involvement. Siltuximab is the only US Food and Drug Administration (FDA) approved treatment. Another IL-6 receptor antibody tocilizumab is approved in Japan. Reports suggest that relapses occur upon cessation of therapy. For iMCD patients who do not respond to IL-6 blockade therapy, various second-line treatment can be considered, and it is challenging to recommend one therapy over another. These options include corticosteroids, rituximab, thalidomide, lenalidomide, bortezomib, cyclosporine, sirolimus, interferon, and others. The ubiquitin-proteasome pathway plays an active role in neoplastic growth. Bortezomib inhibits the ubiquitin-proteasome pathway by blocking the activity of the 26 S proteasome, disrupting multiple downstream signaling pathways in the cellular and bone marrow microenvironment, inducing apoptosis and inhibiting cell cycle progression, angiogenesis, cell adhesion and proliferation [21]. As siltuximab isn’t available in China, we used bortezomib-based chemotherapy, and after seven doses of VCD (Bortezomib, Cyclophosphamide, and Dexamethasone), the patient is in remission.

## Data Availability

The datasets used and analyzed during the current study are available from the corresponding author on reasonable request.

## Abbreviations

iMCD: idiopathic multicentric Castleman disease
IgG4-RD: immunoglobulinG4-related disease
PET/CT: positron emission tomography-CT
^18^F-FDP: 18 F-fluorodeoxyglucose
GLB: globulin
TP: total protein
HHV8: human herpes virus-8
ECOG PS: Eastern Cooperative Oncology Group Performance Status
eGFR: estimated glomerular filtration rate
